# Targeting PCNA in Cancer: A Paradigm Shift from Static Inhibition to Dynamic Network Modulation

**DOI:** 10.32604/or.2026.079988

**Published:** 2026-07-16

**Authors:** Shijia Lu, Yanmin Wang, Han Zhang, Mengjia Yan, Mengdan Sang, Jinle Wang, Huaying Du, Jinwen Sima, Yiran Zhen, Xue Yang, Yutong Zhang, Hongwei Zhou

**Affiliations:** 1 School of Life Science and Technology, North Henan Medical University, Xinxiang, China; 2 School of Medical Laboratory, North Henan Medical University, Xinxiang, China; 3 Basic Medical College, North Henan Medical University, Xinxiang, China

**Keywords:** Proliferating cell nuclear antigen, targeted protein degradation, artificial intelligence, precision therapy, tumor microenvironment, synthetic lethality, clinical translation

## Abstract

Proliferating Cell Nuclear Antigen (PCNA) is a core protein in DNA replication and repair. Its functional dysregulation drives tumorigenesis and therapeutic resistance, making it a critical anticancer target. However, the fundamental conflict between PCNA’s indispensable “guardian” function in normal cells and its hijacked “accomplice” role in cancer cells constitutes the central challenge for targeted intervention: how to eradicate tumors while avoiding severe toxicity to normal tissues. This review aims to systematically review the latest advances and translational dilemmas in the field of PCNA-targeted therapy. It outlines various intervention strategies, including small-molecule inhibitors, proteolysis-targeting chimeras, post-translational modification interference, and synthetic lethality approaches, analyzing their potential and limitations in preclinical research. The review focuses on dissecting key bottlenecks hindering clinical translation, such as the selectivity dilemma, delivery barriers, and resistance evolution. Concurrently, it critically examines how cross-disciplinary technologies—including artificial intelligence, spatiotemporal regulation, and synthetic biology—offer novel ideas to address these bottlenecks, while clarifying that most remain in early exploratory stages. By synthesizing progress, challenges, and future directions, this article provides a framework to inform the development of highly selective and translatable PCNA-based anticancer strategies.

## 1 Introduction

The development and progression of malignant tumors are closely associated with genomic instability [1] and sustained DNA replication stress [2]. Current mainstream therapies still have limitations: conventional radiotherapy and chemotherapy, due to their lack of selectivity, cause severe damage to normal tissues while killing tumors and may accelerate the development of resistance [3]; meanwhile, targeted therapies are often constrained by tumor heterogeneity and rapid adaptive resistance [4].

Against this backdrop, Proliferating Cell Nuclear Antigen (PCNA) has emerged as a highly promising yet challenging new anticancer target, owing to its central, integrative role in DNA replication, repair, and the cell cycle. PCNA forms a homotrimeric ring that acts as a dynamic platform at the replication fork, coordinating numerous DNA metabolism proteins, and is crucial for maintaining genomic stability [5]. This very positioning constitutes both the core paradox and the opportunity for PCNA-targeted therapy: it is an essential “guardian of the genome” required for the survival of all normally proliferating cells, yet in cancer cells, its function is hijacked to support malignant proliferation and survival, becoming an “accomplice in oncogenesis” [6]. This “dual role” presents a unique window for intervention but also poses a formidable translational challenge—how to achieve highly selective killing of cancer cells while avoiding unacceptable toxicity to normal tissues [7]. In recent years, a deeper understanding of the PCNA network and the convergence of interdisciplinary technologies such as artificial intelligence and synthetic biology are driving a paradigm shift in this field from “static inhibition” towards “dynamic network modulation”. These technologies provide powerful tools for deciphering PCNA’s complex interactions and developing new strategies, although the reliability of their predictions and designs ultimately depends on high-quality data, rigorous validation, and experimental confirmation [8].

**Figure 1:**
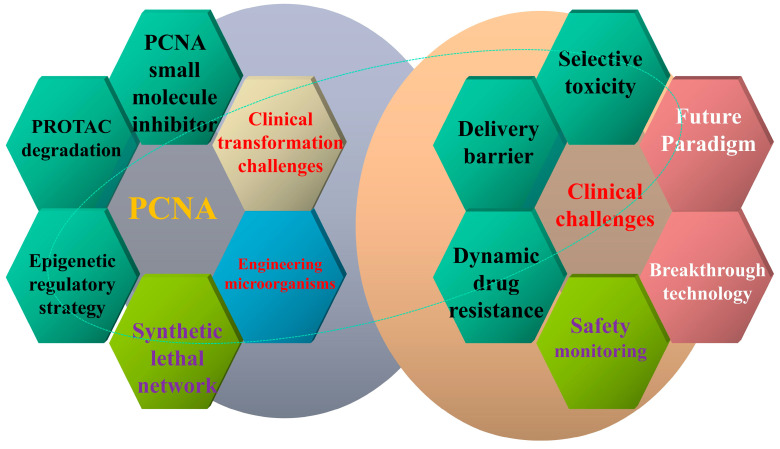
**Schematic of the PCNA-targeted therapy strategy spectrum, translational challenges, and future paradigms.** The PCNA is positioned as the central hub for intervention. Current targeting strategies (**left**) and the key challenges to their clinical application (**right**) converge at the core issue of clinical translation. To address these bottlenecks, the future paradigm points toward integrated, dynamic intervention systems enabled by technologies such as engineered therapeutic microbes. The diagram outlines the logical pathway from mechanistic strategies to clinical application and the innovative routes needed to bridge the translational gap. Abb: Proliferating Cell Nuclear Antigen (PCNA).

This article aims to systematically review the latest advances and core challenges in PCNA-targeted therapy (Fig. 1). This study will outline the main intervention strategies, with a focus on analyzing emerging cutting-edge, interdisciplinary technologies designed to overcome bottlenecks in selectivity, delivery, and resistance. Throughout, this study maintains a critical perspective, scrutinizing the strength of evidence, technological limitations, and translational hurdles. Finally, this article will explore pragmatic future development pathways, with the goal of advancing this promising target from a scientific concept towards a translatable therapy.

## 2 PCNA Targeting Strategies

### 2.1 Small-Molecule Interface Inhibitors

PCNA small-molecule inhibitors primarily target the hydrophobic pocket near the inter-domain connecting loop (IDCL) of its homotrimeric ring. This region is a critical binding site for numerous interacting proteins containing the PCNA-interacting protein box (PIP-box) or AlkB homologue 2 PCNA-interacting motif (APIM). These inhibitors function by dynamically and competitively binding to this site, thereby regulating the assembly of DNA replication and repair complexes [9].

**Figure 2:**
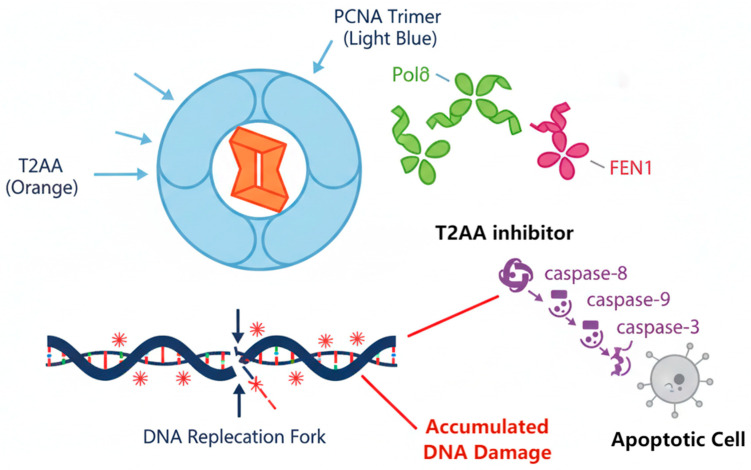
**Schematic model of the mechanism of action for the PCNA inhibitor T2AA.** The small molecule T2AA competitively occupies the central hydrophobic groove of the PCNA trimer (light blue), displacing key replication/repair proteins like FEN1 and DNA polymerase δ. This inhibits PCNA’s scaffold function, blocks translesion synthesis, and causes lethal DNA damage accumulation. The resulting apoptotic cascade is initiated through sequential activation of caspase-8, caspase-9, and caspase-3, leading to tumor cell death. Abb: flap endonuclease 1 (FEN1); T2 amino alcohol (T2AA).

T2AA is an early-reported PCNA small-molecule inhibitor. By mimicking the hydrophobic properties of interacting proteins, it specifically binds to the IDCL region of PCNA, thereby competitively blocking the interaction between PCNA and proteins such as flap endonuclease 1 (FEN1) and DNA polymerase delta (Polδ), and inhibiting the translesion synthesis (TLS) pathway (Fig. 2) [10]. In preclinical studies using osteosarcoma U2OS cells, T2AA, as the first reported non-peptidic small-molecule inhibitor of PCNA/PIP-box interactions, exhibited a biochemical inhibitory activity with an IC_50_ of approximately 1 μM. This compound effectively disrupts the interaction between PCNA and DNA Polδ on cellular chromatin, thereby inhibiting DNA replication, causing S-phase cell cycle arrest, and inducing DNA replication stress [11]. Further studies indicate that T2AA can bind to PCNA in a bivalent mode, with its binding sites located not only at the PIP-box binding cavity but also near the monoubiquitination site Lys-164. This binding mode enables it to inhibit the interaction between monoubiquitinated PCNA and the TLS polymerase η. Functionally, T2AA delays the repair and reactivation of reporter plasmids containing cisplatin-induced interstrand crosslinks. When used in combination with cisplatin, T2AA enhances cisplatin-induced DNA double-strand break formation by inhibiting interstrand crosslink repair. It also promotes the co-localization of phosphorylated ATM and 53BP1 into foci and upregulates phosphorylated BRCA1. This synergistic effect ultimately translates into stronger cytotoxicity; compared to cisplatin treatment alone, the combination of T2AA and cisplatin reduces the clonogenic survival of cancer cells [12]. These data collectively indicate that T2AA, by targeting both PCNA and its monoubiquitinated form, interferes with key DNA damage tolerance and repair pathways, thereby sensitizing cancer cells to chemotherapeutic agents like cisplatin.

**Figure 3:**
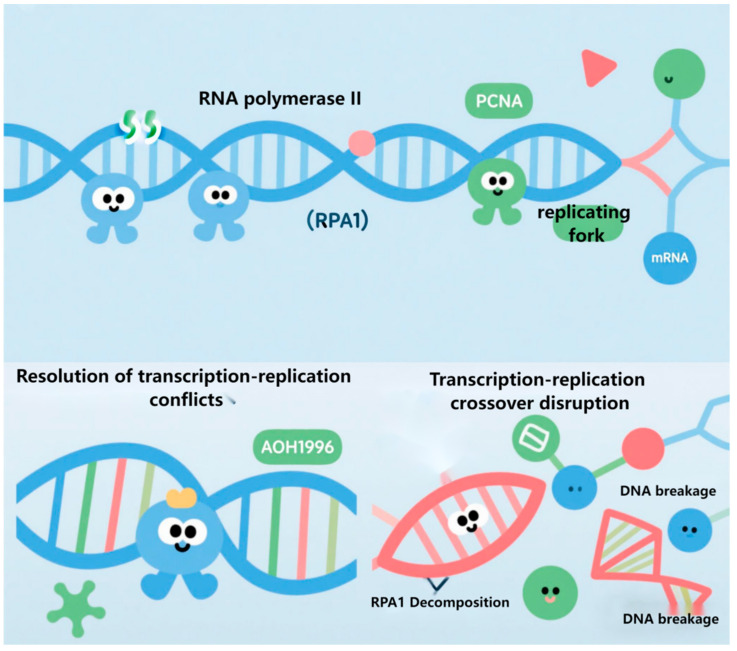
Mechanism of Action Model for the Next-Generation PCNA inhibitor AOH1996: selective killing of tumor cells via stabilized TRCs. The schematic contrasts normal TRC resolution with the drug’s mechanism. AOH1996 binds PCNA and stabilizes its aberrant interaction with RNAP II, effectively sequestering PCNA at transcription sites. This disrupts normal TRC resolution, leading to unresolved R-loop accumulation, RPA1 complex destabilization, and consequent DNA double-strand breakage. This transcription-dependent mechanism preferentially targets cancer cells with high replicative and transcriptional activity, underpinning the compound’s selective anti-tumor effect. Abb: transcription-replication conflicts (TRCs); replication protein A (RPA1); RNA polymerase II (RNAP II).

AOH1996 is a new generation, highly selective PCNA inhibitor. It employs a bidentate key-and-lock binding mode, simultaneously occupying both the PIP-box groove and the adjacent APIM motif-binding region. This unique binding drives the formation of an abnormally stable complex between PCNA and the RNA polymerase II subunit RPB1, leading to irresolvable transcription-replication conflicts, which in turn trigger R-loop accumulation and DNA double-strand breaks (DSBs) (Fig. 3) [13].

In preclinical studies, AOH1996 exhibited broad-spectrum anti-proliferative activity across various cancer cell lines. Notably, in head and neck squamous cell carcinoma (HNSCC) models, AOH1996 effectively inhibited tumor cell proliferation, invasion, and induced apoptosis. Its anti-tumor effects extend beyond direct tumor cell killing, significantly suppressing cancer stem cell (CSC) properties. This is evidenced by a reduction in the proportion of ALDH-high expressing cells, inhibition of tumor sphere formation capacity, and downregulation of stem cell markers such as BMI1, SOX2, ALDH1, and MYC. *In vivo*, AOH1996 monotherapy significantly suppressed the growth of HNSCC xenograft tumors [14]. In pancreatic ductal adenocarcinoma (PDAC) models, the combination of AOH1996 with a KRAS inhibitor demonstrated potent synergistic anti-tumor effects in both KRAS G12C and G12D mutant models. The combination induced cell cycle arrest and apoptosis, and significantly inhibited tumor growth in both tumor organoids and *in vivo* models, with no significant toxicity, such as body weight loss observed [15].

Furthermore, in HNSCC models, AOH1996 reshaped the tumor immune microenvironment towards a more inflammatory state, increasing CD8^+^ T cell infiltration. This remodeling synergized with anti-PD-1 immunotherapy, significantly enhancing therapeutic efficacy. Preclinical safety evaluations indicated that at doses effective in inhibiting tumor growth, AOH1996 did not cause significant side effects in experimental animals, demonstrating a favorable therapeutic window [13]. Based on this compelling preclinical data, AOH1996 has progressed to a Phase I clinical trial for advanced solid tumors.

T2AA and AOH1996 represent the evolution of PCNA-targeting small-molecule drugs from “proof-of-concept” to “optimized candidates”. Research on T2AA established the feasibility of targeting the PCNA-protein interaction interface, though its own drug-like properties require further optimization. AOH1996 demonstrates excellent selectivity and a therapeutic window in preclinical models; however, its “outstanding potential” in overcoming acquired resistance and activating systemic anti-tumor immune responses awaits more in-depth mechanistic exploration and validation in *in vivo* models.

### 2.2 Conformationally Engineered Peptides Targeting PCNA

Given that PCNA’s interactions with numerous partner proteins rely on short linear motifs that are highly conserved through evolution, researchers have developed novel peptide antagonists based on these motifs themselves. Strategies targeting the APIM have achieved significant progress. Structural biology studies reveal that APIM peptides adopt an amphipathic (possessing both hydrophilic and hydrophobic regions) conformation favored by their characteristic β-turn structure. This allows their hydrophobic core to efficiently embed into the PCNA trimer’s groove, which also binds the PIP-box, thereby competitively blocking endogenous protein binding [9].

**Figure 4:**
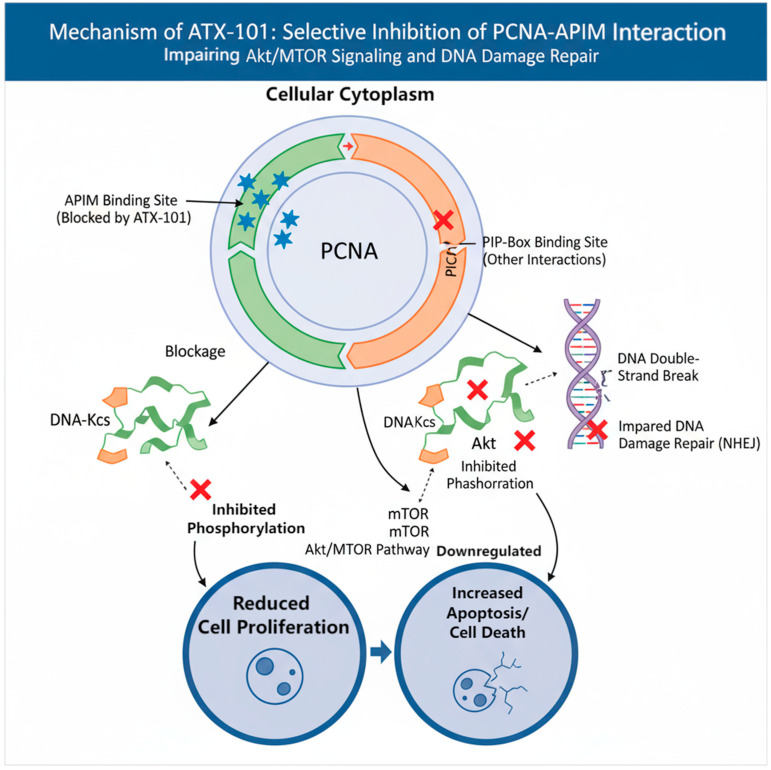
Schematic of the molecular mechanism by which ATX-101 inhibits the Akt/mTOR signaling pathway and DNA damage repair via specific blockade of PCNA-APIM interactions. The PCNA-targeting peptide ATX-101 selectively occupies the APIM-binding site on PCNA, blocking interactions with APIM-containing client proteins. This dual blockade suppresses the Akt/mTOR signaling pathway and impairs DNA double-strand break repair via the non-homologous end joining pathway, leading to reduced tumor cell proliferation and increased apoptosis. Abb: non-homologous end joining (NHEJ); mammalian target of rapamycin (mTOR); Ak strain transforming (Akt).

ATX-101 is a cell-penetrating APIM peptide drug targeting PCNA. Its mechanism of action lies in specifically interfering with PCNA’s scaffolding function during cellular stress responses [16]. By binding to the APIM motif on PCNA, it competitively blocks PCNA’s interaction with numerous partner proteins containing either APIM or non-canonical PIP-boxes. These proteins play critical roles in DNA damage repair, cell signaling transduction, and metabolic regulation (Fig. 4) [17]. Studies show that ATX-101 can downregulate the Akt/mTOR and DNA-PKcs signaling pathways. It also disrupts PCNA’s function as a scaffold for metabolic enzymes (such as ENO1, 6PGD, GAPDH), thereby inhibiting primary metabolism like glycolysis and the pentose phosphate pathway, ultimately inducing cancer cell apoptosis [18,19].

Preclinical studies have confirmed the broad-spectrum antitumor activity of ATX-101. In glioblastoma (GBM) models, ATX-101 demonstrated antiproliferative effects against a panel of human GBM cell lines and patient-derived glioma-initiating cells (GICs). Its sensitivity was independent of PCNA protein levels or the mutational status of genes such as p53, PTEN, and MGMT. When combined with radiotherapy (RT), ATX-101 significantly increased levels of the DNA damage marker γH2AX, promoted DNA fragmentation, and induced apoptosis, exhibiting a strong radiosensitizing effect. *In vivo*, ATX-101 monotherapy or its combination with RT strongly inhibited tumor growth in both subcutaneous and orthotopic GBM xenograft models [19]. Furthermore, studies in multiple myeloma (MM) found that cells with higher intrinsic levels of proteasome and endoplasmic reticulum (ER) stress, upregulated ribosomal gene expression, and lower NAD+/NADH levels were more sensitive to ATX-101. ATX-101 treatment further exacerbated ER stress, reduced primary metabolism, and lowered the glutathione redox couple ratio (GSH/GSSG) [18].

A separate first-in-human Phase I clinical trial (NCT04814875) evaluated the safety, pharmacokinetics, and preliminary efficacy of ATX-101 in patients with advanced solid tumors. Twenty-five heavily pretreated patients received weekly intravenous infusions of ATX-101 at doses of 20, 30, 45, and 60 mg/m^2^. ATX-101 exhibited a favorable safety profile, with no dose-limiting toxicities observed and the maximum tolerated dose not reached. The primary drug-related adverse events were infusion-related reactions (occurring in 64% of patients), manifesting as pruritus, urticaria, and rash, mostly mild to moderate in severity, and manageable by interrupting or slowing the infusion rate. ATX-101 was rapidly cleared from the bloodstream, with an elimination half-life of less than 30 min across all dose levels. Among the 20 patients evaluable for efficacy, although no objective tumor responses were observed, the disease control rate reached 70%, indicating clinical activity in this advanced patient population [16,20].

Based on this preclinical and clinical data, a Phase Ib/IIa proof-of-concept study evaluating ATX-101 in combination with platinum-based chemotherapy in patients with platinum-sensitive ovarian cancer has been initiated.

The preclinical data for ATX-101 validate the feasibility and unique advantages of APIM-based engineered peptides for treating refractory brain tumors like glioblastoma, particularly their high blood-brain barrier penetrability. However, peptide-based drugs generally face inherent challenges such as poor pharmacokinetic stability (rapid degradation by endogenous proteases), low tissue delivery efficiency, and relatively high production costs [21]. Although its combination with radiotherapy demonstrated “unprecedented” survival benefits in preclinical models, this description warrants cautious interpretation; its potential to translate into efficacy for human patients awaits rigorous validation in clinical trials [22]. Currently, this strategy remains in the preclinical research stage. The key to its further development lies in enhancing the *in vivo* stability of the peptides through chemical modifications such as cyclization or incorporation of non-natural amino acids, and in developing more efficient delivery systems to ultimately realize its clinical translational value. The characteristics of PCNA small-molecule and peptide inhibitors are summarized in Table 1.

**Table 1:** Summary of characteristics of small molecule and peptide-based inhibitors of PCNA.

Category	Representative Molecule	Structural/Combination Pattern Characteristics	Interaction with PCNA	Current Research Stage
Small molecule interface inhibitors	T2AA	Non-peptide small molecules that simulate the hydrophobic properties of PIP-box proteins	Specifically binds to the IDCL region of the PCNA trimer, competitively occupying the binding pocket of the PIP-box protein	Preclinical study
Small molecule interface inhibitors	AOH1996	Small molecules employing the “double-gear lock-key” combination mode	Simultaneously occupying the PIP-box binding groove on PCNA and the adjacent APIM motif binding region	Phase I clinical trial (for advanced solid tumors)
Conformationally engineered peptide antagonists	ATX-101	Cell-penetrating peptides designed based on APIM motifs	By mimicking endogenous ligands through its APIM motif, it binds to the APIM binding site on PCNA with high affinity	Phase I clinical trial has been completed (NCT04814875), and Phase Ib/IIa study is currently underway

Abb: Proliferating Cell Nuclear Antigen (PCNA); inter-domain connecting loop (IDCL); PCNA-interacting protein box (PIP-box); AlkB homologue 2 PCNA-interacting motif (APIM); T2 amino alcohol (T2AA).

### 2.3 PROTAC-Mediated PCNA-Targeted Degradation Strategy

Proteolysis-targeting chimera (PROTAC) technology represents a revolutionary therapeutic paradigm. It operates through an event-driven pharmacological mechanism, bringing the protein of interest (POI) into proximity with an E3 ubiquitin ligase, thereby inducing polyubiquitination and subsequent proteasomal degradation of the POI. This results in the complete removal of the target protein, rather than merely inhibiting its function. This catalytic mechanism allows a single PROTAC molecule to be recycled and degrade multiple target protein molecules, offering a novel possibility for targeting traditionally “undruggable” targets [23,24].

However, the first-generation PROTACs (PROTAC 1.0) face numerous inherent challenges in clinical translation [24], including poor cell membrane permeability, suboptimal pharmacokinetic properties, potential off-target toxicity, and the complex “hook effect”. Especially for a protein like PCNA, which performs essential functions in normal cells, achieving tumor tissue-selective degradation is critical [25]. This has driven the development of “PROTAC 2.0”, whose core focus lies in utilizing novel E3 ligase ligands, optimizing linker chemistry, and developing conditionally activated strategies to expand the range of degradable targets and improve tissue selectivity [23]. Innovation in delivery systems is key to overcoming the bioavailability bottleneck of PROTACs. For example, self-assembling peptide-derived PROTAC nanoparticles can achieve tumor-targeted delivery and sustained PD-L1 protein degradation, showing promise in cancer immunotherapy [26]. Similar nanodelivery strategies, such as lipid nanoparticles and polymeric micelles, are widely studied to protect PROTAC molecules from rapid clearance and enhance their accumulation at tumor sites, thereby advancing their clinical translation [27].

Despite significant challenges, PROTAC technology has achieved a milestone clinical advancement. The oral estrogen receptor PROTAC degrader Vepdegestrant (ARV-471), in a Phase III clinical trial for treating ER+/HER2-advanced breast cancer, significantly prolonged median progression-free survival compared to the standard treatment fulvestrant in patients harboring ESR1 mutations. This demonstrates the efficacy and clinical translational feasibility of PROTACs in patients, providing valuable experience and confidence for the development of PROTACs targeting other proteins, including PCNA [28]. In summary, by continuously optimizing molecular design to enhance selectivity and integrating advanced nanodelivery systems to improve pharmacokinetics, the PCNA-targeting PROTAC strategy holds promise to overcome current challenges. Ultimately, this catalytic degradation modality may be translated into a precise therapy for refractory cancers.

### 2.4 Synthetic Lethality Strategies Targeting the PCNA Post-Translational Modification Network

The function of PCNA is also precisely regulated by a variety of post-translational modifications (PTMs). This dynamic modification network directly influences the choice and efficiency of DNA damage repair pathways [29]. Targeting specific PCNA-modifying enzymes or “readers” has emerged as a novel strategy to interfere with tumor DNA repair and induce a “synthetic lethal” effect.

Structural biology studies provide the foundation for precise intervention. Cryo-electron microscopy analysis has revealed a specific hydrogen-bonding network formed between the K164 site on PCNA and the ubiquitination E2/E3 complex Rad6/Rad18, elucidating the structural mechanism initiating PCNA monoubiquitination [30,31]. Building on this, research found that inhibitors of the deubiquitinating enzyme USP7 (e.g., HBX 41108) can stabilize the translesion synthesis polymerase Polη, thereby positively enhancing the monoubiquitination level of PCNA. This provides, for the first time, mechanistic evidence that the “USP7-Polη-PCNA ubiquitination” axis is a key node regulating the DNA damage response [32].

More translationally promising is targeting the SUMOylation of PCNA. SUMOylation inhibitors (e.g., TAK-981) can specifically inhibit the SUMOylation of PCNA at its K107/K110 sites [33]. In BRCA1-deficient breast cancer preclinical models, this inhibition disrupts the co-localization of PCNA with the key homologous recombination (HR) repair protein RAD51 on chromatin, leading to a collapse of HR function. This induces a strong “synthetic lethal” effect in tumor cells while having minimal impact on normal cells [34]. This mechanism shares homology with observations in KRAS mutant models, where the combination of a SUMO inhibitor and a MEK inhibitor synergistically disrupts the RAD51-BRCA1 repair pathway, further validating the therapeutic value of targeting PCNA SUMOylation in specific genetic contexts [35].

The association between different PCNA modification states and therapeutic response is becoming increasingly clear, and detection technologies are advancing towards higher resolution and dynamic monitoring. Taking K29 acetylation as an example, studies first identified its association with drug-resistant phenotypes in cancer stem cell populations using high-throughput techniques like “single-cell glycomics” [36,37]. Subsequently, “dynamic modification profile monitoring” technology was developed, aiming to track changes in a patient’s PCNA modification profile in real-time. Its area under the curve (AUC) for predicting disease progression can reach 0.94, providing a potential basis for timely clinical treatment adjustment [38].

Although strategies targeting the PCNA modification network demonstrate significant “synthetic lethal” potential in preclinical models with DNA repair defects (like BRCA mutations), their claimed “breakthrough efficacy” is primarily derived from preclinical research and urgently awaits validation in human clinical trials. The clinical translation of this strategy faces multiple core challenges: First, the various PTMs on PCNA form a complex and interactive dynamic network; intervention targeting a single modification may be compensated for by other alternative pathways [39]. Second, processes like SUMOylation and acetylation are fundamental and widespread cellular activities, so inhibitors may carry off-target risks and cause unforeseen toxicity to normal tissues [40]. Finally, there is a lack of validated biomarkers to accurately screen patient populations likely to benefit and to monitor treatment efficacy. For instance, the clinical utility of dynamic modification profiles like PCNA K29 acetylation still requires rigorous validation [41]. Currently, leading SUMOylation inhibitors have entered early-stage clinical research. Their ultimate success will depend on establishing a sufficiently wide therapeutic window in specific patient populations and effectively overcoming the tumor’s inherent compensatory resistance mechanisms.

### 2.5 Synthetic Lethality Strategies Targeting the PCNA Interaction Network

PCNA plays a crucial role in maintaining genomic stability through its specific interactions with DNA repair proteins, such as the core mismatch repair (MMR) complex MutLα. Targeting these precise protein-protein interaction interfaces provides a highly specific strategy to induce a “synthetic lethal” effect in tumors with DNA repair deficiencies.

Structural biology studies have elucidated the molecular basis: the 721QRLIAP motif at the C-terminus of the PMS2 subunit binds to the inter-domain connecting loop of PCNA via hydrophobic interactions and a key hydrogen-bonding network. This interface is an allosteric regulatory hotspot essential for MMR function [42]. Based on this structural insight, researchers designed the targeting peptide PMS2-PIP-6, which mimics the PCNA-binding motif of PMS2 to competitively block the formation of the endogenous MutLα-PCNA complex. Preclinical validation shows that disrupting this interaction in MLH1-deficient colorectal cancer models severely impairs MMR function. In organoid models, PMS2-PIP-6 reduced MMR efficiency to approximately 8% of wild-type levels, leading to replication fork collapse [43]. Particularly important, this specific disruption of MMR generated a potent synthetic lethal effect in the context of DNA repair deficiency, increasing tumor cell sensitivity to the PARP inhibitor Olaparib by 5.7-fold. This provides a new strategy for overcoming PARPi resistance and treating microsatellite instability-high (MSI-H) tumors [43,44].

Although this strategy is conceptually attractive, it currently remains in the early preclinical research stage, and its clinical translation faces multiple challenges: The applicable patient population may be limited to tumors with MLH1/PMS2 deficiency. As a peptide drug, PMS2-PIP-6 faces classic hurdles such as poor membrane permeability, low *in vivo* stability, and delivery difficulties. Its efficacy within complex tumor microenvironments and its synergistic potential with other therapies still require systematic evaluation. Future directions lie in developing small-molecule inhibitors with superior drug-like properties and exploring their application potential in broader contexts of DNA repair deficiency.

## 3 Translational Challenges and Breakthrough Strategies

Despite demonstrating significant potential in preclinical research, PCNA-targeted anticancer strategies face multiple challenges in clinical translation. These hurdles encompass core issues such as insufficient targeting selectivity, limited drug delivery efficiency, the evolution of resistance mechanisms, and a narrow therapeutic window. The root causes lie in the unique structural plasticity of PCNA and the complexity of the regulatory network involving its ubiquitination and modifications.

### 3.1 The Selectivity Dilemma

PCNA is indispensable for maintaining genomic stability in normal cells, which constitutes the core “selectivity dilemma” of its targeted therapy—how to effectively kill tumors while maximizing the protection of healthy tissue. Current small-molecule inhibitors face distinct selectivity challenges and risk profiles depending on their mechanisms of action, which directly impact their therapeutic window and potential clinical application direction.

T2AA works by competitively occupying the IDCL region of PCNA, broadly inhibiting multiple DNA repair pathways, including TLS. Its primary selectivity risk stems from the inhibition of replication factors like Polη/δ, which may impair the homeostasis of rapidly proliferating normal tissues, such as intestinal stem cells. Preclinical data show that in ovarian cancer models, the combination of T2AA and cisplatin can enhance efficacy while reducing bone marrow toxicity [11]. This suggests its potential indication may be in combination with platinum-based chemotherapy to sensitize tumors and reduce toxicity, potentially offering an advantage in tumors with active TLS pathways. The corresponding clinical consideration involves closely monitoring toxic side effects in normally proliferating tissues like the gastrointestinal tract and exploring the optimal combination dose and schedule.

AOH1996 functions by inducing TRCs and R-loop accumulation. Its remarkable tumor selectivity (>30-fold) in preclinical solid tumor models has yielded encouraging tumor regression rates (up to 83.7%) and a relatively wide safety window [7]. However, its core risk lies in the fact that persistently unresolved R-loops may cause genomic instability, potentially driving tumor evolution and acquired resistance. Therefore, its potential indication might lean towards exploiting its unique mechanism for treating tumors exhibiting high replication stress or ATR pathway activation. Key clinical preventative measures will include developing biomarkers to monitor genomic instability and designing rational combination strategies to prevent or overcome resistance.

In summary, T2AA and AOH1996 represent two different paradigms for addressing the PCNA selectivity dilemma: the former focuses on synergy with existing chemotherapy but requires strict monitoring of its potential toxicity to normal tissues; the latter, while showing significant monotherapy activity, carries the non-negligible risk of inducing genomic instability. Future breakthroughs in overcoming this core dilemma hinge on systematically transforming the “selectivity” challenge into a “precision” opportunity. To this end, it is crucial to establish mechanism-based therapeutic index models to quantify the real therapeutic window of different inhibitors. Building on this, the future lies in using molecular subtyping to precisely identify the patient subgroups most likely to benefit, achieving “mechanism-directed therapy”. Ultimately, it is also necessary to explore and optimize novel strategies, such as intermittent dosing, to alleviate the long-term stress on normally proliferating tissues caused by continuous PCNA targeting, thereby achieving a fundamental balance between efficacy and safety.

### 3.2 Innovation in Delivery Systems

PCNA-targeted therapies, especially macromolecular formulations, face severe delivery challenges in clinical translation. While small-molecule drugs possess good cell membrane permeability, their penetration in solid tumors is often limited by dense biological and physical barriers such as the extracellular matrix and high interstitial fluid pressure, hindering effective distribution to the tumor core [45]. For central nervous system tumors, the blood-brain barrier (BBB) presents a nearly insurmountable obstacle, suppressing the brain delivery efficiency of most systemically administered drugs to extremely low levels (typically below 0.2% for conventional chemotherapeutics) [46].

**Figure 5:**
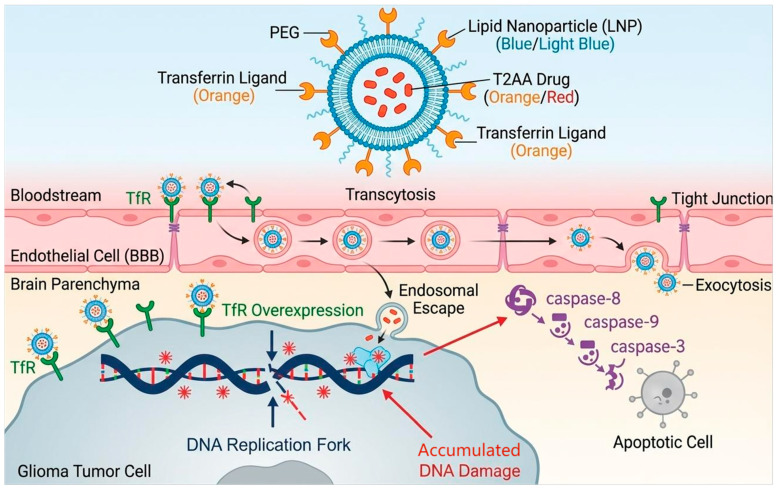
**Schematic of the mechanism of action for transferrin receptor-targeted lipid nanoparticles delivering the PCNA inhibitor T2AA to treat glioblastoma.** The schematic illustrates the journey of T2AA-loaded LNPs from the bloodstream to the induction of tumor cell apoptosis. Surface-conjugated transferrin ligands enable binding to TfR on the blood-brain barrier endothelium, mediating transcytosis into the brain parenchyma. Following internalization by glioma cells, LNPs escape from acidic endosomes to release T2AA into the cytoplasm. The inhibitor enters the nucleus, binds PCNA at the replication fork, disrupts DNA repair, and causes lethal DNA damage. This triggers a sequential caspase cascade (caspase-8/9/3), culminating in apoptotic cell death. Abb: Lipid nanoparticles (LNPs); transferrin receptor (TfR).

To overcome these barriers, researchers have developed a transferrin receptor (TfR)-targeted lipid nanoparticle (LNP) system. This system achieves receptor-mediated transcytosis across the BBB by surface-modifying the nanoparticles with a TfR ligand, exploiting the high expression of TfR on brain capillary endothelial cells [47]. Simultaneously, by optimizing the nanoparticle’s hydrodynamic diameter (typically under 100 nm) and surface PEGylation, the system extends blood circulation time while also accumulating at the tumor site via the enhanced permeability and retention (EPR) effect (Fig. 5) [48,49].

In preclinical studies, for instance, in a mouse model of lung cancer brain metastasis, this TfR-targeted LNP system demonstrated significant delivery advantages. Quantitative imaging and pharmacokinetic analysis showed that, compared to non-targeted, non-specific LNPs, the TfR-targeted design increased drug accumulation in brain metastases by 7.3-fold. Ultra-high performance liquid chromatography-tandem mass spectrometry (UHPLC-MS/MS) detection confirmed a drug concentration in the brain parenchyma of 0.82 ± 0.11 μM, with a brain tissue-to-plasma drug concentration ratio significantly exceeding the therapeutic threshold, proving its efficient ability to cross the BBB and target intracranial lesions [50].

However, this highly efficient targeted delivery strategy introduces new safety considerations. TfR is widely expressed on various cell types, and the endocytosis it mediates may intersect with complex cellular signaling pathways. Some preclinical studies suggest that sustained activation of the TfR pathway may potentially upregulate the expression of proteins like matrix metalloproteinase-9 (MMP-9), which is associated with tumor invasion and metastasis [50]. Therefore, while acknowledging its breakthrough in delivery efficiency, a cautious assessment of the long-term safety of this strategy is essential. Future optimization directions include developing environmentally responsive “stealth” PEG shells that shed only in the acidic tumor microenvironment. This aims to enhance targeted accumulation at the tumor site while reducing nonspecific uptake and potential off-target effects in normal tissues.

The TfR-targeted LNP system represents an important preclinical-stage breakthrough in addressing brain delivery challenges. However, its positioning as a “breakthrough advancement” requires a dual perspective: on one hand, it dramatically increases drug exposure in brain tumors in animal models; on the other hand, its potential for off-target effects and signaling perturbations necessitates more comprehensive preclinical safety pharmacology evaluations. This technology currently remains in the preclinical development stage. Its successful translation will depend on the ability to further optimize targeting specificity in subsequent studies, validate the optimal balance between efficacy and safety in large animal models, and ultimately transition to human clinical trials.

### 3.3 Evolution of Drug Resistance: Mechanisms, Heterogeneity, and Counterstrategies

PCNA-targeted therapy, like other targeted therapies, inevitably faces the core challenge of tumor adaptive resistance. The development of resistance is a multi-layered, dynamically evolving process involving genetic heterogeneity, epigenetic remodeling, the tumor stem cell microenvironment, and adaptive mutations in the PCNA protein itself.

#### 3.3.1 Genetic Heterogeneity and Activation of Compensatory Repair Pathways

Tumors exhibit high intratumoral genetic heterogeneity. Single-cell transcriptomics analysis reveals a significant disparity in PCNA expression levels within the tumor (i.e., a super-dispersed distribution) [51,52]. This heterogeneity may be associated with the compensatory activation of the DNA damage response (DDR). Studies have found that under therapeutic pressure, certain tumor cell subpopulations may upregulate the ATM/ATR signaling pathway (manifested as increased phosphorylation levels of key proteins), accompanied by the overexpression of polymerase theta (Polθ), thereby activating alternative DNA repair pathways, such as TLS and microhomology-mediated end-joining (MMEJ), to circumvent the replication crisis triggered by inhibited PCNA function [53]. However, whether this association constitutes a direct causal relationship still requires further validation through lineage tracing and functional gene knockout experiments.

#### 3.3.2 Epigenetic Remodeling and Dysfunctional Replication Stress Surveillance

Dysregulation of epigenetic control is another key driver of resistance evolution. Research has observed that in acquired resistance models, the level of histone H3 lysine 27 acetylation (H3K27ac) modification in the PCNA gene promoter region is increased and shows spatial co-localization with the remodeling of chromatin super-enhancers [54]. This change in epigenetic state is accompanied by a significant upregulation of WEE1 kinase expression. WEE1 is a key kinase regulating the G2/M cell cycle checkpoint and replication fork stability. Its burst-like high expression may interfere with normal replication stress surveillance, allowing cells to enter mitosis with incompletely repaired DNA damage, thereby promoting genomic instability and survival [55]. It is important to note that whether the enrichment of H3K27ac is the “cause” or the “effect”, and whether other epigenetic drivers independent of PCNA exist, are current gaps in research.

#### 3.3.3 Unique Adaptive Mechanisms of the CSC Microenvironment

CSCs are considered the root of drug resistance and recurrence. Evidence indicates that in glioblastoma, the CD133^+^ CSC subpopulation can modify PCNA at serine 129 (Ser129) via O-GlcNAcylation [56]. Computational studies based on molecular dynamics simulations predict that this modification may alter the local conformation of PCNA through an allosteric effect, leading to an increase in the binding free energy (i.e., weaker binding) of small-molecule inhibitors. This prediction aligns with experimental observations: the half-maximal inhibitory concentration (IC_50_) of AOH1996 for CD133^+^ cells increased by approximately 300% compared to parental cells [57]. This suggests that targeting PCNA glycosylation or CSC metabolism may be a novel strategy to overcome resistance.

#### 3.3.4 Acquired Mutations in PCNA and Conformational Selection Resistance

Long-term therapeutic pressure may directly select for PCNA mutants. In a preclinical study, a point mutation (G178D) located near the PCNA trimer interface was selected through serial passaging (up to the 10th generation) of a patient-derived xenograft (PDX) model under drug pressure [58]. Crystal structure analysis and molecular modeling suggest that this mutation may disrupt the local hydrogen-bonding network essential for trimer stability, causing a change in the geometry of the drug-binding pocket (calculated to reduce its volume by approximately 18%). This would lower the binding affinity of inhibitors, resulting in “conformational selection” resistance [59]. However, the frequency and clinical significance of such mutations in patient tumor samples, especially prior to receiving PCNA-targeted therapy, remain unknown.

To address the complex resistance mechanisms emerging in PCNA-targeted therapy, researchers are seeking breakthroughs from two directions: synergistic intervention and drug innovation. Regarding synergistic strategies, based on the principle of synthetic lethality, preclinical studies have found that combining PCNA inhibitors with PARP inhibitors can significantly enhance efficacy, for instance, increasing tumor growth inhibition by 4.7-fold in specific models [60]. Overall, the key to overcoming resistance and achieving durable efficacy lies in: the early identification of resistance biomarkers based on PCNA modifications or mutations; the design of sequential or combination therapies targeting different resistance mechanisms; and the use of AI-assisted design for next-generation drugs capable of adapting to target evolution. The successful implementation of these comprehensive strategies will ultimately determine the depth and breadth of the clinical translation of PCNA-targeted therapies.

### 3.4 Safety Challenges and Precision Intervention Strategies in Clinical Translation

PCNA is indispensable in the normal cell cycle. This dictates that the core bottleneck for the clinical translation of PCNA-targeted therapies is the narrow therapeutic window—the dose effective for killing tumors is too close to the dose toxic to normal tissues, especially rapidly proliferating hematopoietic stem cells and germ cells. Overcoming this challenge requires the development of precision intervention strategies capable of distinguishing tumors from normal tissues.

Among the strategies explored to precisely improve the therapeutic index, two preclinical approaches have shown potential. One is tumor-selective epigenetic silencing, which utilizes the hTERT promoter specifically activated in cancer cells to drive a CRISPR-dCas9/KRAB system, delivered via lipid nanoparticles, aiming to selectively silence genes like FEN1 at the tumor site [61]. The other is a tumor-microenvironment-responsive immuno-modulatory PROTAC. This design links an EXO1-targeting PROTAC to an anti-PD-L1 antibody via a disulfide bond cleavable within the tumor, intending to synchronously release the degrader and the immune checkpoint inhibitor in the reductive tumor microenvironment, achieving local protein degradation and immune activation [62]. Although studies have reported that the combined application of these strategies in specific preclinical models could expand the therapeutic index by approximately 6.8-fold, these data must be interpreted with caution. This result originates from a highly controlled, specific model whose generalizability is unproven. Therefore, this numerical value absolutely cannot be equated with having overcome the core safety barriers of clinical translation. It merely indicates a promising research direction and is still far from solving the complex safety challenges of human application.

These strategies represent cutting-edge concepts for enhancing safety through “biological discrimination” and “pathophysiological discrimination”. However, they are all currently in the early preclinical research stage and face severe translational challenges (key strategies for PCNA-targeted therapy and their characteristics are summarized and compared in Table 2). First, their delivery efficiency and specificity remain unresolved. For instance, the *in vivo* targeted delivery and long-term safety of the CRISPR-LNP system need optimization, and the hTERT promoter is not absolutely tumor-specific as it also shows activity in some normal stem cells. Second, pharmaceutical and regulatory hurdles for complex products are substantial. Novel biologics like antibody-PROTAC conjugates pose great difficulty in manufacturing, quality control, and understanding their complex pharmacokinetic/pharmacodynamic relationships. Most critically, preclinical models cannot accurately simulate human complexity. There is high uncertainty about whether these strategies can achieve the envisioned “high efficacy, low toxicity” goal in humans. Therefore, it is premature to label them as “breakthrough” or “firewall” technologies. They are more appropriately viewed as proof-of-concept tools for validating the principle of “precision detoxification”. Their true clinical feasibility depends on the future ability to systematically overcome the aforementioned challenges in more clinically relevant models and, ultimately, to validate them through rigorous human trials.

**Table 2:** Overview and characteristics comparison of core strategies for PCNA targeted therapy.

Strategy Categories	Representative	Mechanism of Action	Core Advantage	Main Challenges
Small molecule interface inhibitors	AOH1996, T2AA	Block the PIP-box/APIM interaction interface, induce replication-transcription conflict, or inhibit TLS	High oral bioavailability, rapid clinical conversion	Selective toxicity, potential drug resistance
Peptide/peptide-like antagonist	ATX-101	Competitively inhibit the interaction between PCNA and repair proteins	High affinity, penetration of the blood-brain barrier	Poor metabolic stability, low delivery efficiency
PROTAC degrader	Con1-SPROTAC, RS80E	Induce ubiquitination and degradation of PCNA	Overcoming drug resistance, capable of targeting “undruggable” proteins	Large molecular weight, difficult for delivery to solid tumors
Epigenetic regulation	TAK-981 (SUMO inhibitor)	Inhibit PCNA SUMOylation, disrupt the repair complex	Synthetic lethal	Off-target effects, complex regulatory network
Synthetic lethality application	PMS2-PIP-6 peptide	Target the PCNA-PMS2 interaction, disrupt MMR	Highly selective for killing tumors with DNA repair defects	Limited applicability

Abb: translesion synthesis (TLS); Proteolysis-targeting chimera (PROTAC); mismatch repair (MMR).

## 4 Integration of Artificial Intelligence and Synthetic Biology in PCNA-Targeted Therapy Exploration

With the development of interdisciplinary fields, the domain of PCNA-targeted therapy is exploring new paradigms beyond traditional inhibition. Tools like artificial intelligence, synthetic biology, and optogenetics provide new possibilities for understanding and intervening in the dynamic network of PCNA. However, it is crucial to view these cutting-edge technologies with caution at their current stage: most remain in the proof-of-concept or early preclinical research phase, and their clinical translation path, reliability, and ultimate feasibility await rigorous evaluation. This section will critically examine these emerging technologies, highlighting their prospects while clearly pointing out their methodological transparency, level of validation, and the substantial obstacles they currently face.

### 4.1 Application of AI in Drug Discovery: Progress, Transparency, and Limitations

#### 4.1.1 Data Integration and Dynamic Modeling

Researchers have constructed an all-atom resolution model, PCNA-DeepModel v1.0, by integrating publicly available PCNA structural data, compound activity data, and internally generated single-cell epigenomics (scATAC-seq) data, combined with large-scale molecular dynamics simulations [63,64]. This model aims to simulate the four-dimensional dynamics of PCNA-ligand interactions. It is important to note that its predictive accuracy is significantly constrained by the quality and coverage of the training data, the accuracy of the force field parameters, and its outputs still require validation by wet-lab experiments.

#### 4.1.2 AI-Driven Molecular Generation and Optimization

In the optimization of lead compounds, reinforcement learning was applied to design the second-generation inhibitor AOH2035. Studies report that the optimized molecule achieved a binding free energy (ΔG) of −12.3 kcal/mol and a molecular surface complementarity index of 0.82. It also demonstrated a higher blood-brain barrier penetration rate (3-fold higher than its predecessor) in a glioblastoma PDX model [65]. While these are positive preclinical data, the core questions regarding its clinical feasibility—namely, its pharmacokinetics, safety, and ultimate efficacy in humans—remain to be answered by future clinical trials.

#### 4.1.3 Drug Repurposing and Preliminary Clinical Signals

The application of a graph neural network-based drug repurposing platform (DRP-PCNA) provides a reference case from computation to clinic. By analyzing large-scale drug-target interaction maps, the platform predicted and experimentally confirmed that the antifungal drug terbinafine could bind to the SUMOylation interface of PCNA and induce replication fork collapse [66]. A subsequent Phase II clinical trial interim analysis reported an observed objective response rate (ORR) of 34.8% in a specific patient cohort. This example is often cited to illustrate the value of AI-driven R&D. However, it requires cautious interpretation: First, this success rate is highly dependent on the quality and completeness of the training data. Second, the 34.8% ORR is an interim result that needs confirmation in the final analysis and larger-scale trials. Finally, this discovered “correlation”—the association between terbinafine’s efficacy and its predicted action on PCNA—still requires more mechanistic studies to establish a direct causal relationship and to rule out other potential target(s) of action.

AI undoubtedly accelerates the discovery and optimization process of PCNA-targeted drugs and has generated promising preclinical candidate molecules and repurposing clues. However, the assessment of its “revolutionary” impact must be based on a full understanding of its methodological limitations, the rigor of validation, and the uncertainties of clinical translation. Currently, AI’s primary role in the PCNA field remains that of a powerful auxiliary tool, not an autonomous system replacing traditional biological research.

### 4.2 Exploration of Spatiotemporally Precise Regulation Technologies

To overcome the potential normal tissue toxicity from systemically targeting PCNA, researchers are endeavoring to develop intervention tools with spatiotemporal precision, aiming to confine activity to the tumor locale. Most of these technologies are currently in the proof-of-concept or early preclinical research stage. Their core goal is to achieve a paradigm shift from “continuous inhibition” to “on-demand activation”.

#### 4.2.1 Optogenetic Regulation Platform

The Opto-PCNA optogenetic system is representative of this direction. This platform is constructed via genetic engineering to create a CRY2olig-PCNA fusion protein, utilizing the light-controlled dimerization properties of the photosensitive protein CRY2 and the E3 ligase substrate receptor CRBN. In *in vitro* experiments, laser irradiation at a specific wavelength can induce rapid binding between CRY2 and CRBN (response time can reach the nanosecond scale), thereby precisely controlling the ubiquitination and degradation of PCNA in space (theoretical resolution ~200 μm) and time [67]. This provides a powerful “molecular scalpel” for studying PCNA function in complex cell populations or 3D culture models. However, its translation to *in vivo* therapeutic application faces significant challenges, including how to safely and efficiently deliver the optogenetic components to tumor cells *in vivo*, and the limited penetration depth of visible light in biological tissues.

#### 4.2.2 Environmentally Responsive PROTAC Technologies

Another strategy involves developing “smart” PROTACs capable of sensing tumor microenvironment features. This mainly includes two categories.

Light-Activatable PROTACs (e.g., LIGHT-PROTAC): This approach involves attaching a “photocage” (e.g., o-nitrobenzyl) to the PROTAC molecule to mask its activity. Only upon irradiation with a specific wavelength is the “cage” removed, restoring the PROTAC’s activity and thereby achieving spatially localized protein degradation at the irradiation site [68]. This technology is similarly limited by light penetration in tissues and is currently primarily applicable to superficial or endoscopically accessible lesions.

pH-Responsive PROTACs (e.g., pcPROTAC): This strategy exploits the typically more acidic nature of tumor tissue compared to normal tissue (the acidic microenvironment). pcPROTAC molecules incorporate pH-sensitive moieties such as benzimidazole, which undergo conformational changes in acidic environments, thereby activating their degradation function [69]. A preclinical study reported that in an A549 lung cancer metastasis model, the pH-responsive design increased drug accumulation or target degradation efficiency at the tumor site by approximately 10.3 ± 1.2-fold compared to a non-responsive control [70].

Although spatiotemporally precise regulation technologies are designed in principle to improve the selectivity of PCNA-targeted therapy and hold broad prospects, it is essential to clearly recognize that the vast majority of this research remains in the foundational or early preclinical exploratory stage. Their clinical translation faces severe challenges. The primary difficulty is the reliable delivery and uniformity of the control signal; achieving uniform and controllable drug activation within solid tumors, which exhibit significant heterogeneity, is extremely difficult, whether relying on light or pH changes. Secondly, the safety and immunogenicity of the systems themselves cannot be ignored. For example, optogenetic components may trigger immune responses, and UV light irradiation carries potential carcinogenic risks. Furthermore, complex pharmaceutical development is a major obstacle; environmentally responsive PROTACs are more complex than traditional PROTACs in terms of synthesis, stability, and pharmacokinetics. Therefore, it is premature to describe these technologies as realized “intelligent biological switches” or a “paradigm shift”. They are currently more appropriately viewed as proof-of-concept tools for validating the principle of “spatiotemporal control”. Their ultimate success in clinical application depends entirely on the future ability to systematically address these fundamental challenges in more complex disease models that better approximate the human condition.

### 4.3 Exploratory Applications of Synthetic Biology in PCNA Intervention

Synthetic biology provides a novel toolbox for developing new anticancer therapies by designing programmable biological systems. In the field of PCNA-targeted therapy, researchers are exploring the use of engineered cells, viruses, or bacteria to construct “intelligent” systems capable of sensing the tumor microenvironment and responding. These studies are highly forward-looking, aiming to validate the principle and feasibility of a new therapeutic paradigm: “sensing-decision-execution”. However, the research must clearly state that the vast majority of these systems are in the early proof-of-concept stage of preclinical research, and their complexity, safety, and ultimate efficacy in humans remain uncertain.

#### 4.3.1 Engineered Immune Cells

Engineered macrophages are designed to carry a CRISPR-dCas9 gene silencing system controlled by a tumor metabolite-responsive promoter [54]. The design logic is: when the cells are recruited to the lactate-enriched tumor microenvironment, the promoter is activated, driving the expression of the dCas9/KRAB complex, which subsequently silences the PCNA gene [71]. Preclinical studies report that this design can reduce PCNA expression in tumor-associated macrophages in *in vitro* and simple *in vivo* models, with observed changes in the associated cytokine profile [72]. Its core challenges are: precise tumor homing, long-term survival, and functional maintenance of engineered immune cells *in vivo* are difficult to control; the CRISPR system carries potential off-target effects and immunogenicity risks; and lactate, as a signal, is distributed in both tumors and some normal tissues, making its specificity not absolute.

#### 4.3.2 Transcriptionally Targeted Oncolytic Virus

The oncolytic virus CRAd-PCNA-TRAIL is designed by inserting a key viral replication gene controlled by the PCNA gene promoter into its genome. This design aims for the virus to selectively replicate only in proliferating tumor cells with high PCNA expression, thereby lysing tumor cells and releasing tumor-associated antigens, while the virus also expresses the pro-apoptotic factor TRAIL to enhance killing [73]. A preclinical study reported that in a specific immunocompetent mouse model, combining this virus with a PD-1 antibody resulted in a high tumor complete response rate (reported in the study as 60%–70%) [74]. This requires cautious interpretation: this excellent efficacy was achieved in a controlled mouse model with a consistent genetic background, whose immune system differs from humans; the “PCNA promoter” is also active in some normal proliferating cells, and the virus may attack these tissues; its safety, particularly the potential risk of systemic viral dissemination, must be strictly evaluated in more advanced animal models.

#### 4.3.3 Engineered Bacterial Therapy

The engineered Escherichia coli Nissle 1917 (EcN-PCNA) strain is modified to carry a genetic circuit capable of expressing PCNA-intervening factors, integrating both a hypoxia-responsive module and a quorum-sensing module [75]. The design logic is as follows: after intravenous injection, the bacteria accumulate in the hypoxic regions of the tumor; when the bacterial population within the tumor reaches a certain threshold, a “lysis circuit” is activated, releasing the therapeutic payload. This aims to achieve tumor-localized treatment while reducing the risk of systemic bacteremia. Preclinical studies in a mouse liver cancer model demonstrated colonization of the engineered bacteria at the tumor site and showed some antitumor effects [76,77]. This approach faces fundamental challenges: the biosafety risk of live bacterial therapy is extremely high, potentially triggering uncontrollable infections or a cytokine storm; the colonization efficiency of bacteria in patients with an intact immune system may be far lower than in immunodeficient mouse models; the actual delivery efficiency and persistence of the therapeutic payload within the tumor remain significant hurdles.

**Figure 6:**
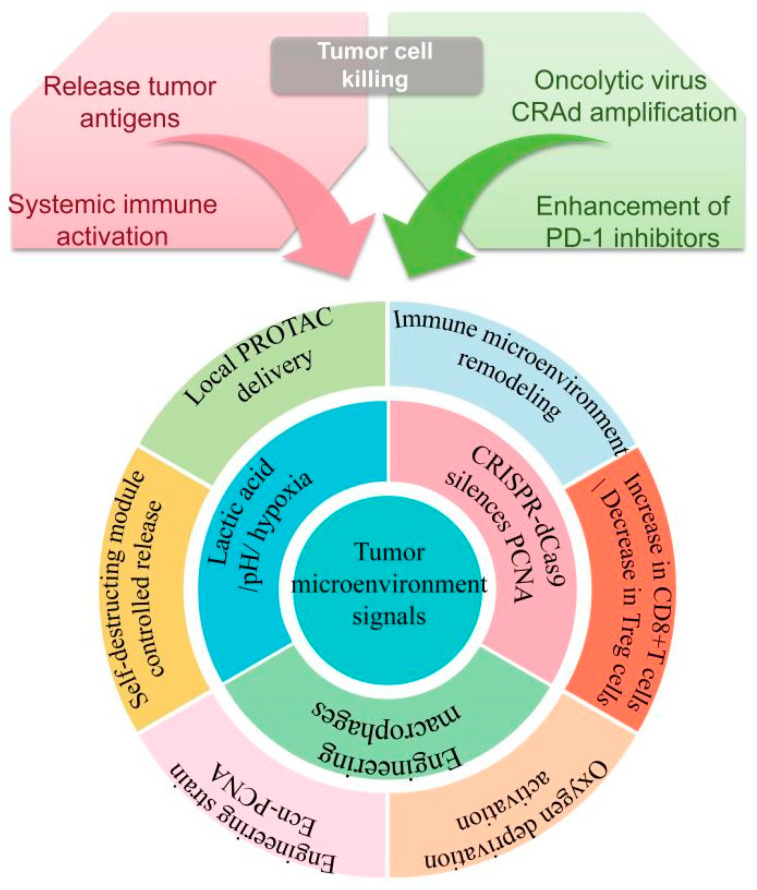
Schematic of a synthetic biology-driven intelligent closed-loop therapeutic system. This schematic illustrates a synthetic biology-based platform for multi-level tumor targeting. Engineered carriers, responsive to tumor microenvironmental cues, deliver localized interventions such as PROTACs and CRISPR-Cas9 to silence PCNA, thereby inhibiting proliferation and inducing tumor cell death. The released tumor antigens, combined with microenvironmental reprogramming, convert an immunosuppressive tumor into an immunogenic state. Oncolytic viruses further amplify tumor lysis and immune stimulation. This localized response primes a systemic anti-tumor immune response, which can be enhanced with immune checkpoint inhibitors, resulting in coordinated local and systemic tumor control. Abb: Proteolysis-targeting chimera (PROTAC).

Although synthetic biology strategies bring unprecedented potential for logical and spatial control in PCNA intervention, outlining a vision for “sense-and-respond” intelligent therapy, the claimed substantial improvements in therapeutic window often originate from highly idealized early animal experiments. These findings exhibit a significant gap with the complexity of human disease contexts and should not be directly extrapolated. The clinical translation path for this technology is long and fraught with obstacles: Safety is the primary barrier, as off-target effects, escape, or uncontrolled proliferation of live engineered organisms could have fatal consequences, and the regulatory pathway is extremely stringent; Human complexity is a fundamental challenge, as the tumor microenvironment, immune system, and microbiome background in humans are far more complex than in mouse models, and many designs effective in animals may fail in humans; Manufacturing and quality control also pose major difficulties, as the production, quality assurance, storage, and administration standards for live biologic products are extremely complex and costly. Therefore, the current primary value of synthetic biology in PCNA intervention lies in proof-of-principle and conceptual exploration, far from indicating imminent clinical translation (Fig. 6). It is premature to describe it as “rewriting the therapeutic paradigm” or having established an “intelligent closed loop”. Future research must focus on systematically addressing fundamental issues of safety, specificity, and controllability in models that more closely resemble the clinical setting.

### 4.4 Multi-Omics Integration: Exploration from Molecular Subtyping to Dynamic Monitoring

Advances in single-cell multi-omics and novel biosensing technologies provide high-resolution tools for dissecting the dynamic regulatory network of PCNA in complex systems. These technologies aim to correlate the molecular features of PCNA with therapeutic response, thereby driving more precise patient stratification and efficacy monitoring. However, the clinical utility of these analytical platforms is still in the early validation stages, and their claimed efficacy must be carefully evaluated in conjunction with specific experimental contexts and methodological limitations.

#### 4.4.1 Single-Cell Multi-Omics Analysis

Single-cell multi-omics platforms, by combining antibody-mediated protein detection with high-throughput single-cell transcriptome sequencing, enable the correlation of PCNA protein expression, its specific post-translational modifications, and global transcriptional states at the individual cell level [78]. One study applied this technology to analyze a triple-negative breast cancer cohort, identifying a subpopulation of tumor cells with a high PCNA K164 ubiquitination signal. *In vitro* experiments showed that this subpopulation exhibited significantly increased sensitivity to PARP inhibitors compared to other subpopulations [79]. Further molecular analysis suggested that this subpopulation may be generally characterized by homologous recombination repair deficiency, providing a plausible mechanistic hypothesis for the observed sensitivity difference. It is important to clarify that this association is derived from retrospective analysis of a specific patient cohort, and its generalizability needs validation in larger-scale, prospective cohorts. Furthermore, whether high K164 ubiquitination is the direct cause of PARP inhibitor sensitivity or is associated with other co-occurring genomic alterations requires more rigorous functional experiments to establish causality.

#### 4.4.2 Nanopore-Based Dynamic Monitoring Technology

Nanopore-sensing-based technology attempts to directly and dynamically detect different modified forms of PCNA in body fluids. This technology utilizes high-affinity peptide probes that bind to specific PCNA modifications. The identification and quantification of the modification states are achieved by measuring the characteristic current changes caused when the peptide-target complex translocates through a nanopore [80]. A proof-of-concept study reported that in the longitudinal analysis of plasma samples from patients with advanced breast cancer, samples from treatment responders exhibited a stronger K164 ubiquitination signal (with an average intensity approximately 3.7 times that of baseline or non-responders), whereas samples from patients with progressive disease primarily showed a K29 acetylation signal. The study further reported that, within this limited analyzed sample set, the sensitivity for predicting disease progression based on this modification profile change was 210% higher compared to a traditional circulating tumor DNA (ctDNA) detection method [81]. This data must be interpreted with caution: the comparison result is highly dependent on the specific ctDNA detection method chosen, the thresholds set, and the particular patient population. The conclusion of a “210% improvement” requires rigorous validation in independent, larger patient cohorts to assess its reproducibility and general applicability.

Single-cell multi-omics and nanopore monitoring technologies provide promising research directions for achieving dynamic analysis of PCNA, jointly outlining a blueprint from high-dimensional molecular subtyping to potential real-time monitoring. However, these technologies currently serve primarily as powerful research tools, remaining a significant distance from mature clinical diagnostic applications. Single-cell technology faces practical challenges such as high cost, limited throughput, complex data analysis, and the difficulty of obtaining high-quality clinical sample suspensions. The biomarker subgroups it identifies must have their predictive value validated in prospective clinical trials. Liquid biopsy technologies, on the other hand, need to address fundamental issues, including the extremely low abundance of the target in plasma, the difficulty in balancing sensitivity and specificity, the lack of standardized protocols, and the challenge of distinguishing PCNA modifications originating from tumor versus normal cells. Therefore, describing the current state of these technologies as having already established a “closed-loop management system” is premature. A more accurate positioning is that they are innovative research tools under active development, capable of generating hypotheses and discovering potential biomarkers. Their ultimate integration into clinical practice depends entirely on future large-scale clinical studies confirming their reliable predictive performance and clinical utility under standardized and cost-effective conditions.

## 5 Conclusions and Future Perspectives

PCNA, as a core hub coordinating DNA replication and repair, has seen considerable progress in its targeted therapy in recent years. A variety of intervention approaches, ranging from traditional small-molecule interface inhibitors, PROTAC-mediated protein degradation, and interference with post-translational modification networks, to synthetic lethality strategies targeting specific DNA repair defects, have continuously demonstrated their scientific feasibility and therapeutic potential in preclinical research. However, this review systematically reveals that the core obstacle to the successful clinical translation of this field does not stem from the absence of a single technology. Instead, it is rooted in the fundamental paradox dictated by PCNA’s biological function itself: the conflict between its indispensable “guardian” role for all proliferating cells and its hijacked “accomplice” role in cancer cells. This contradiction directly leads to the severe “selectivity dilemma”, from which a series of interrelated translational challenges, including delivery efficiency, the evolution of resistance, and a narrow therapeutic window, are derived.

The introduction of interdisciplinary technologies such as artificial intelligence, synthetic biology, and spatiotemporal regulation provides unprecedented new ideas and powerful tools to address the aforementioned bottlenecks. They have greatly deepened our understanding of the dynamic PCNA network and spawned highly imaginative novel intervention strategies, such as environmentally responsive PROTACs and logic-gated engineered bacteria. However, it is crucial to maintain a clear understanding that the vast majority of these cutting-edge strategies are still in the proof-of-concept or early preclinical exploration stage. Their primary current value lies in serving as powerful research tools for elucidating mechanisms, validating hypotheses, and discovering new biomarkers, rather than indicating imminent clinical translation. Any claims of “breakthrough efficacy” or a “paradigm shift” must be built upon a foundation of substantial, rigorous, reproducible follow-up experimental data and strict future validation in clinical trials.

Looking ahead, advancing PCNA-targeted therapy from a promising scientific concept to a clinical reality that benefits patients depends on a pragmatic, rational, and collaborative development path. First, a phased integration and validation strategy should be implemented: prioritize advancing relatively mature candidates with superior drug-like properties into early clinical trials, while positioning high-risk, high-technical-barrier strategies like synthetic biology vectors as long-term exploratory directions. Second, the development of biomarkers and precise patient stratification is key to breaking through the “selectivity dilemma”. It is essential to fully leverage technologies such as single-cell and liquid biopsy multi-omics to prospectively identify and validate biomarkers capable of predicting efficacy and toxicity within clinical trials. This will enable “mechanism-directed therapy”, maximizing therapeutic benefit while controlling risks. Finally, strengthening interdisciplinary and international collaboration is paramount. By establishing international collaborative networks and data-sharing platforms focused on DNA damage response targets, research methodologies can be standardized, clinical trial designs coordinated, and the cross-validation and learning cycles of different strategies accelerated, thereby systematically advancing the entire field towards its ultimate goal.

## Data Availability

Not applicable.
